# A multi-task, multi-stage deep transfer learning model for early prediction of neurodevelopment in very preterm infants

**DOI:** 10.1038/s41598-020-71914-x

**Published:** 2020-09-15

**Authors:** Lili He, Hailong Li, Jinghua Wang, Ming Chen, Elveda Gozdas, Jonathan R. Dillman, Nehal A. Parikh

**Affiliations:** 1 The Perinatal Institute and Section of Neonatology, Perinatal and Pulmonary Biology, Cincinnati Children’s Hospital Medical Center, 3333 Burnet Avenue, MLC 7009, Cincinnati, OH 45229 USA; 2 Department of Pediatrics, University of Cincinnati College of Medicine, Cincinnati, OH USA; 3 Department of Radiology, University of Cincinnati College of Medicine, Cincinnati, OH USA; 4 Department of Electronic Engineering and Computing Systems, University of Cincinnati, Cincinnati, OH USA; 5 Imaging Research Center, Cincinnati Children’s Hospital Medical Center, 3333 Burnet Avenue, MLC 7009, Cincinnati, OH 45229 USA; 6 Department of Radiology, Cincinnati Children’s Hospital Medical Center, 3333 Burnet Avenue, MLC 7009, Cincinnati, OH 45229 USA

**Keywords:** Brain imaging, Magnetic resonance imaging, Machine learning

## Abstract

Survivors following very premature birth (i.e., ≤ 32 weeks gestational age) remain at high risk for neurodevelopmental impairments. Recent advances in deep learning techniques have made it possible to aid the early diagnosis and prognosis of neurodevelopmental deficits. Deep learning models typically require training on large datasets, and unfortunately, large neuroimaging datasets with clinical outcome annotations are typically limited, especially in neonates. Transfer learning represents an important step to solve the fundamental problem of insufficient training data in deep learning. In this work, we developed a multi-task, multi-stage deep transfer learning framework using the fusion of brain connectome and clinical data for early joint prediction of multiple abnormal neurodevelopmental (cognitive, language and motor) outcomes at 2 years corrected age in very preterm infants. The proposed framework maximizes the value of both available annotated and non-annotated data in model training by performing both supervised and unsupervised learning. We first pre-trained a deep neural network prototype in a supervised fashion using 884 older children and adult subjects, and then re-trained this prototype using 291 neonatal subjects without supervision. Finally, we fine-tuned and validated the pre-trained model using 33 preterm infants. Our proposed model identified very preterm infants at high-risk for cognitive, language, and motor deficits at 2 years corrected age with an area under the receiver operating characteristic curve of 0.86, 0.66 and 0.84, respectively. Employing such a deep learning model, once externally validated, may facilitate risk stratification at term-equivalent age for early identification of long-term neurodevelopmental deficits and targeted early interventions to improve clinical outcomes in very preterm infants.

## Introduction

Survivors following very premature birth (i.e., ≤ 32 weeks gestational age)^1,2^ remain at high risk for neurodevelopmental impairments, thereby increasing their risk for poor educational, health, and social outcomes^3,4^. Unfortunately, it may take 2–5 years from birth to identify such high-risk infants. Early, accurate identification, soon after birth, could pave the way for domain-specific, intensive early neuroprotective therapies during a critical window for optimal neuroplasticity. In addition to a variety of clinical prognostic factors, atypical brain connectome has been reported in children who develop adverse neurodevelopmental outcomes, suggesting that brain connectome aberrations could serve as a promising prognostic biomarker^5–7^.

The brain connectome is a comprehensive description of the brain’s structural and functional connections in terms of brain networks^8,9^. Brain connectome patterns are formed during brain maturation and reshaped in cases of prematurity or perinatal brain injury^10^. Recent advances in deep learning techniques have made it possible to extract physiologically meaningful features and reveal new discriminative information from high dimensional connectome data^11,12^. There is a growing interest in developing artificial intelligence neural network approaches to predict neurological deficits using connectome data^13,14^, but their use in preterm populations has been very limited^15,16^. Such models typically require training on large datasets^17,18^, and unfortunately, large neuroimaging datasets are either unavailable or expensive to obtain. Kawahara et al. implemented a data augmentation technique to generate synthetic data in their application of a convolutional neural network framework to predict cognitive and motor outcomes from diffusion tensor imaging derived brain structural connectome^16^.

Much of human learning involves only a few new examples superimposed on extensive prior knowledge^19^. Motivated by how human learn new knowledge, transfer learning^20,21^ focuses on storing knowledge gained from solving problems in one *data-rich* domain and applying it to a *new* problem in another data-scarce domain. Transfer learning represents an important key to solve the fundamental problem of insufficient training data in deep learning^22–27^. We developed a deep transfer learning (DTL) neural network framework for enhancing the classification of whole brain functional connectome in small datasets and demonstrated its utility for detection of autism spectrum disorder (ASD)^28^. More recently, we applied this DTL neural network model for early prediction of cognitive deficits at 2 years corrected age in very preterm infants^29^. We pre-trained the model using 884 subjects from the Autism Brain Imaging Data Exchange-I (ABIDE-I) database^30^ in an unsupervised fashion. Unlike our previous work, in this study, we hypothesize that the knowledge gained from classifying ASD patients versus typically developing controls in children and adults can be transferred to predict neurodevelopmental deficits in infants. To test the hypothesis, we propose a multi-stage DTL strategy by taking advantages of both supervised and unsupervised learning techniques. Briefly, we first pre-train a DNN prototype in a supervised fashion using 884 subjects with annotated labels from ABIDE-I database, then re-train this prototype without supervision using 291 neonatal subjects, and finally fine-tune and evaluate the DNN using 33 very preterm infants who have undergone their neurodevelopment assessments at 2 years corrected age. We then hypothesize that multi-stage DTL will outperform the single-stage DTL for the abnormal neurodevelopmental outcome prediction in very preterm infants.

In addition, instead of only cognitive deficit (single-task), we propose to jointly predict multiple neurodevelopmental deficits (multi-task) in very preterm infants, including cognitive, language, and motor skills at 2 years of age. Rather than training each prediction model independently for a single outcome, we train a joint model that is able to encode the shared representations of brain networks related to different outcomes. We further hypothesize that simultaneous prediction of multiple outcomes (i.e., learning related tasks jointly) can improve the performance compared with the prediction of single outcome independently (i.e., learning each task individually). Moreover, besides brain connectome data, this newly proposed model can also incorporate clinical data that have been previously demonstrated to be prognostic biomarkers for neurodevelopmental outcomes^16^. We finally hypothesize that fusing brain connectome and clinical data will improve prediction performance over using either clinical data only or brain connectome data only. In summary, we propose a multi-task, multi-stage DTL framework using the fusion of brain connectome and clinical data for early joint prediction of abnormal neurodevelopmental (cognitive, language and motor) outcomes at two years corrected age in very preterm infants.

## Methods

### Subjects

This study contains three subject cohorts: a source cohort, an intermediate cohort, and a target cohort. Subjects in the source cohort were from the ABIDE-I repository^30^, which has openly shared processed neuroimaging data from 1,112 subjects, including 539 ASD patients and 573 typically developing controls (age range = 7–64 years, median age = 14.7 years) across 17 independent data sites (https://fcon_1000.projects.nitrc.org/indi/abide/). All ABIDE-I subjects were diagnosed using Autism Diagnostic standardized instruments based on clinical judgment, and/or Autism Diagnostic Observation Schedule, and/or Autism Diagnostic Interview—Revised. Based on the autism severity scores, categorical labels (i.e., ASD and typically developing controls) were applied^31^. The intermediate cohort included 67 prospectively enrolled healthy full-term neonates born between 38 and 42 weeks gestational age (born at Ohio State University Medical Center, Riverside Hospital, or Mount Carmel St. Ann’s Hospital in Columbus, Ohio from September 2014 to August 2015) and 246 prospectively enrolled very preterm infants born at 32 weeks gestational age or younger from five Greater Cincinnati region hospitals including, Cincinnati Children’s Hospital (CCHMC), University of Cincinnati Medical Center, Good Samaritan Hospital, St. Elizabeth’s Healthcare, and Kettering Memorial Hospital from November 2016 to October 2018. The target cohort included prospectively enrolled 58 very preterm infants born at 32 weeks gestational age or younger from Nationwide Children’s Hospital (NCH), Ohio State University Medical Center, Riverside Hospital, and Mount Carmel St. Ann’s Hospital in Columbus, Ohio from December 2014 to April 2016. We excluded infants with confounding medical conditions that are known to be associated with poor outcomes, including any congenital or chromosomal anomalies that affected the central nervous system and infants with cyanotic congenital heart disease. For the healthy term controls, we also excluded infants with significant maternal conditions (e.g., insulin-dependent diabetes or severe preeclampsia), intrauterine drug or alcohol exposure, and history of perinatal distress or other birth-related complications. The Institutional Review Boards of NCH and CCHMC approved the study. Approval at the other hospitals was obtained through research reciprocity agreements that were in place with NCH and CCHMC. We obtained written informed consent from a parent/guardian of every enrolled infant from the intermediate and target cohorts. The study methods were carried out in accordance with the relevant guidelines and regulations.

All very preterm infants in the target cohort received standardized Bayley Scales of Infant and Toddler Development III test in the NCH High-Risk Follow-up Clinic at 2 years corrected age. The standardized Bayley scores used in this study were all corrected by the participants’ age at the assessment. The Bayley-III cognitive, language, and motor scores are on a scale of 40 to 160, with a mean of 100 and standard deviation (SD) of 15. We grouped very preterm infants in the target cohort using a cutoff of 85 into those at high versus low risk for moderate/severe neurodevelopmental deficits^32^. More specifically, a child with a cognitive score of ≤ 85 was considered to have high risk of developing severe/moderate cognitive deficits; similarly a child with a language score of ≤ 85 was considered to have high risk of developing severe/moderate language deficits; and a child with a motor score of ≤ 85 was considered to have high risk of developing severe/moderate motor deficits. Therefore, each child will have three outcome labels according to his/her Bayley-III cognitive, language, and motor scores.

For each very preterm infant in the target cohort, 84 clinical features were retrieved from our electronic medical record system (Epic Systems Corporation; Verona, WI). Clinical features related to six overarching domains, including: (1) maternal demographics (e.g., mothers age, gravida, parity, mother’s highest educational level, etc.); (2) pregnancy complications (e.g., multiple births, diabetes, hypertension, hypothyroidism, etc.); (3) labor and delivery (e.g., rupture of membrane, antenatal steroids, magnesium, etc.); (4) neonatal information at birth (e.g., sex, gestational age, birth weight, etc.); (5) medical history (e.g., oxygen or positive pressure support, surfactant administration, pneumothorax, sepsis, bronchopulmonary dysplasia, etc.); and (6) neonatal information at follow-up (e.g., status of infant, weight, length, etc.). The full list of 84 clinical features is elaborated in Supplemental Table 1.

### MRI acquisition

Full term infants in the intermediate cohort and very preterm infants in the target cohort were scanned on a 3 T MRI scanner (Skyra; Siemens Healthcare) at NCH using a 32-channel phased array head coil. All imaging was performed during natural sleep and without sedation after being fed and swaddled. MRI noise was minimized using Insta-Puffy Silicone Earplugs (E.A.R. Inc., Boulder, CO) and Natus Mini Muffs (Natus Medical Inc., San Carlos, CA). Resting-state functional images were collected using a single-shot echo planar image sequence sensitized to T2* weighted blood oxygenation level dependent (BOLD) signal changes. Acquisition parameters were as follows: repetition time (TR) = 3,000 ms, echo time (TE) = 35 ms, flip angle (FA) = 90°, resolution 2.8 × 2.8 × 3.0 mm^3^. A total of 300 frames were collected in 8:00 min. Anatomical scans were conducted with a 2D T2-weighted fast spin-echo sequence: TR = 9,500 ms, TE = 147 ms, FA = 150°, voxel dimensions 0.93 × 0.93 × 1.0 mm^3^; time 4:09 min.

Very preterm infants in the intermediate cohort were scanned on a 3 T MRI scanner (Ingenia, Philips Healthcare, Best, Netherlands) at CCHMC using a 32-channel head coil. Acquisition parameters were as follows for resting state functional MRI: TR = 1,195 ms, TE = 45 ms, flip angle FA = 55°, resolution 2.5 × 2.5 × 2.5 mm^3^. A total of 400 frames were collected in 8:12 min. Anatomical scans were conducted with a 2D T2-weighted fast spin-echo sequence: TR = 8,300 ms, TE = 166 ms, FA = 90°, voxel dimensions 1.0 × 1.0 × 1.0 mm^3^; time 3:53 min.

ABIDE-I subjects in source cohort were scanned at 17 different data sites. The details of the scanning information to the original project site are readily accessible here: https://fcon_1000.projects.nitrc.org/indi/abide/.

### Resting state fMRI data preprocessing

We employed our neonatal-optimized pipeline^7^ for neonatal resting state fMRI preprocessing using FMRIB Software Library (FSL, Oxford University, UK), Statistical Parametric Mapping software (SPM, University College London, UK) and Artifact Detection Tools (ART, MIT, Cambridge, US). The preprocessing pipeline included operations of anterior commissure—posterior commissure reorientation skull stripping; normalization; spatial smoothing (Gaussian filter with 6 mm kernel); band-pass filtering (0.008 < f < 0.09 Hz); and motion artifact reduction. To mitigate the influence of motion and thus disentangle motion effects from functional connectivity effects, we characterized and modeled noise sources related to motion by nuisance regressors, including (1) motion scrubbing^33^; (2) six rigid body realignment parameters (frame-to-frame estimates of the rotation and translation of the head about three cardinal axes); (3) expansion of other nuisance time series obtained by shifting the originals forward or backward in time, by computing their temporal derivatives; and (4) a set of orthogonal time series computed via principle component analysis over white matter and cerebrospinal fluid^34^.

The resting state functional MRI data from ABIDE-I study were already preprocessed with multiple different pipelines and available from the Preprocessed Connectome Project (https://preprocessed-connectomes-project.org/abide/)^35^. In this study, we only included data that were processed with the Configurable Pipeline for the Analysis of Connectomes (C-PAC)^36^.

### Whole-brain functional connectome construction

A brain connectome is a comprehensive map of neural connections in the brain. Mathematically, a connectome is a graph, representing the brain connectivity (described as a set of edges) between pairs of brain regions of interest (ROI) (described as a set of nodes). The connectome can also be encoded as an adjacency matrix, in which each entry represents the brain connectivity between each pair of ROIs. Ninety ROIs were defined based on an adult and a neonatal automated anatomical labeling atlas^37,38^ for ABIDE-I and our neonatal subjects, respectively. This resulted in a 90 × 90 adjacency matrix symmetric about the diagonal, in which each entry represents the brain functional connectivity between each pair of ROIs. The functional connectivity was defined as the temporal correlation of BOLD signals between spatially apart ROIs^39,40^. This was calculated using functional connectivity toolbox (CONN)^41^. The brain functional connectome of 884 subjects from 17 ABIDE-I data sites were normalized to mitigate site bias using the established method in a prior study^42^.

### Multi-task multi-stage deep transfer learning model

#### Overview

**Figure 1:**
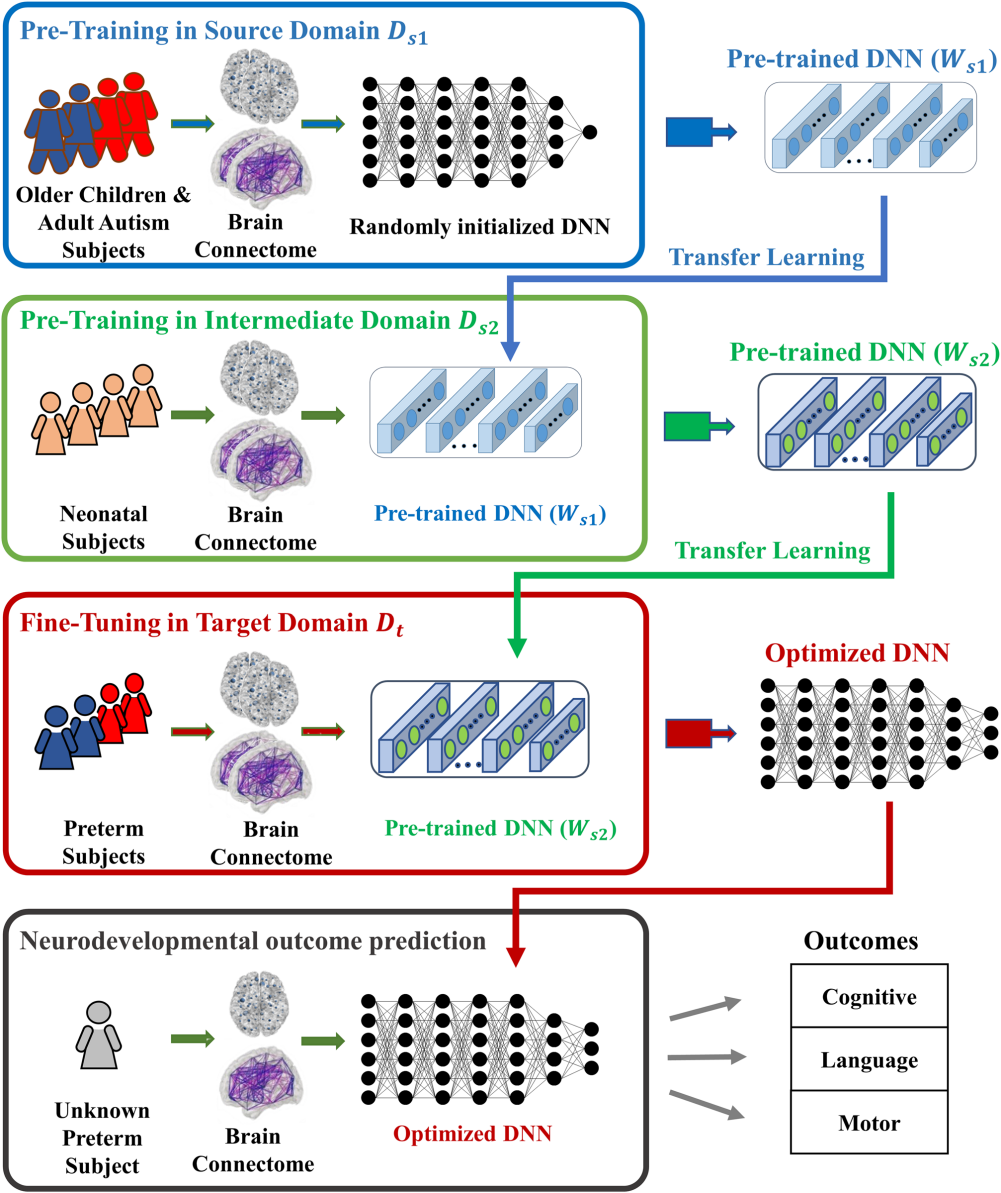
An overview of proposed multi-task, multi-stage deep transfer learning framework for early joint prediction of neurodevelopmental outcomes at 2-years corrected age in very preterm infants.

**Figure 2:**
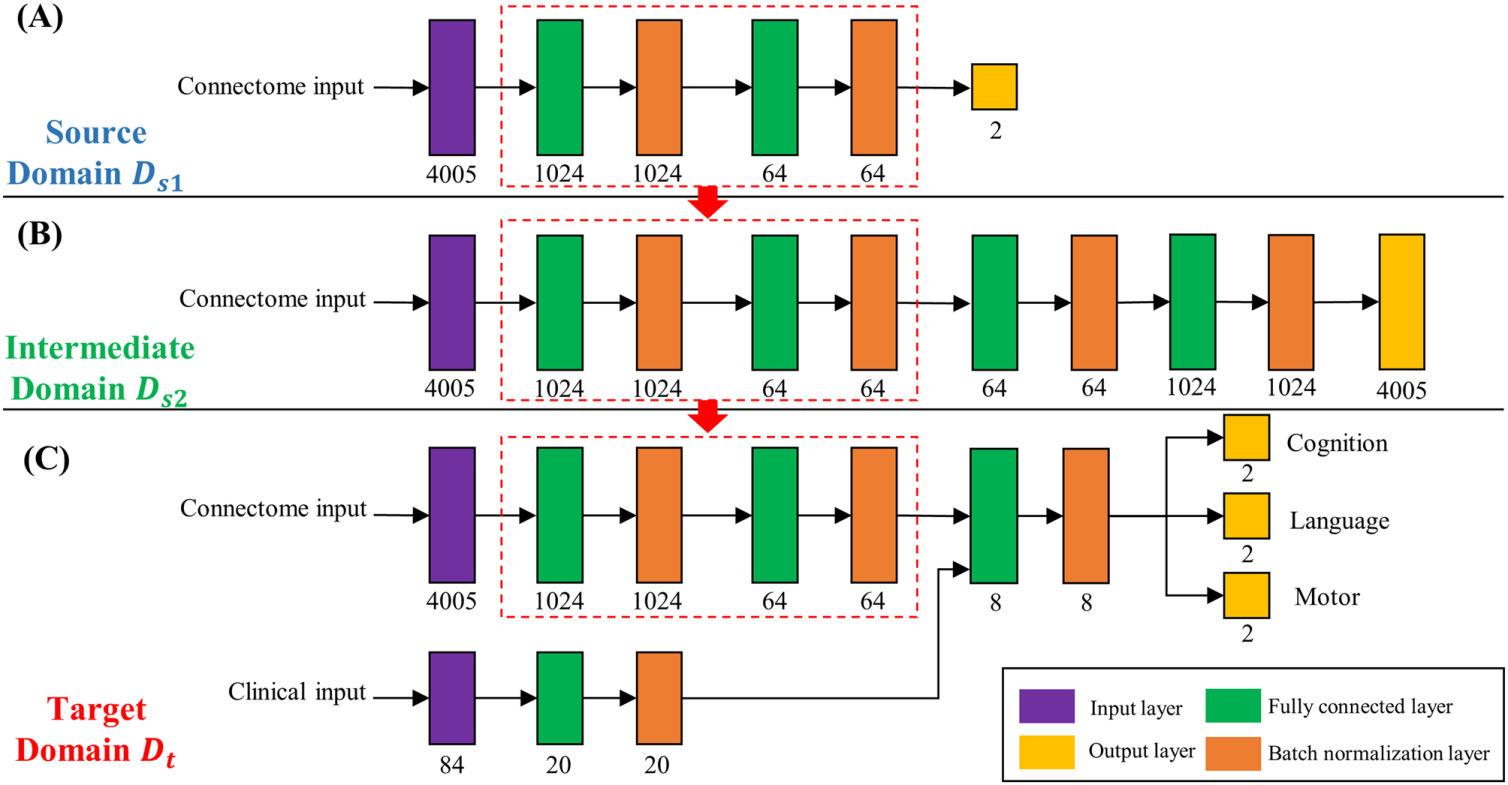
Detailed model architectures in source domain (**A**), intermediate domain (**B**), and target domain (**C**). Layer types are color-coded. Purple: input layer; green: fully connected layer; orange: batch normalization layer; yellow: output layer. The number of nodes in each layer are listed below individual layers. Red color dotted boxes highlight the transferred/reused neural network layers between consecutive domains.

An overview of the proposed multi-task, multi-stage DTL model is shown in Fig. 1. Our learning task is to predict multiple neurodevelopmental (cognitive, language and motor) deficits based on very preterm infants brain connectome data in target domain ${D}_{t}$. Instead of direct transfer the knowledge of brain connectome patterns learned from source domain ${D}_{s1}$ (referred to as single-stage DTL), our proposed multi-stage DTL approach aims to benefit from learning brain connectome patterns in both source domain ${D}_{s1}$ and intermediate domain ${D}_{s2}$, respectively, by reusing neural network weights ${W}_{s1}$ and ${W}_{s2}$. The detailed model designs in source, intermediate, and target domains are shown in Fig. 2, respectively.

More specifically, the proposed model includes four modules: (1) supervised pre-training in source domain ${D}_{s1}$ using ABIDE-I subjects with annotated labels (Fig. 1, top blue panel). We first pre-train a deep neural network (DNN) prototype (Fig. 2A) to learn the representation of brain networks from a group of autism and control subjects. The architecture of this prototype was optimized to achieve the state-of-the-art performance^42^; (2) unsupervised pre-training in intermediate domain using neonatal subjects without annotated labels (Fig. 1, green panel). We adapt the pre-learned brain connectome from module 1 by reusing the neural network weights. To better represent neonatal brain connectome, we re-train the pre-weighted DNN prototype (Fig. 2B) using a group of neonatal (term and preterm) subjects; (3) model fine-tuning in target domain using 33 very preterm infants with annotated labels (Fig. 1, red panel). After the first two modules, prior knowledge about neonatal brain connectome patterns is now encoded in the second-stage DNN prototype from module 2 and ready to be further customized using our target population (i.e., very preterm infants). After the fine-tuning, we will have an optimized model (Fig. 2C) for the (4) joint neurodevelopmental outcome prediction (Fig. 1, bottom gray panel).

#### Stage 1: supervised training in source domain

Given *m* training samples in ABIDE-I dataset from source cohort, {(${\varvec{x}}^{1} ,y^{1}$), …$\left( {{\varvec{x}}^{i} ,y^{i} } \right)$, ….,$({\varvec{x}}^{m} ,y^{m}$)}, where ${\varvec{x}}^{i}$ is the *i-th* input and $y^{i}$ is its corresponding label. We constructed a 5-layer DNN (Fig. 2A), which takes randomly initialized ${\mathbf{W}}_{0}$. A rectified linear unit activation function was used in hidden nodes, and a softmax unit was utilized in the output layer and minimizes the following cross-entropy objective function to obtain pre-trained network weights ${\mathbf{W}}_{s1}$

$$J\left( {{\mathbf{W}}_{s1} ,{\varvec{b}}{|}{\mathbf{W}}_{0} } \right) = - \frac{1}{m}\mathop \sum \limits_{i = 1}^{m} y^{i} \log \left( {p\left( { y^{i} {|}{\varvec{x}}^{i} , {\mathbf{W}}_{0} ;{\mathbf{W}}_{s1} ,{\varvec{b}}} \right)} \right) + \left( {1 - y^{i} } \right)\log \left( {1 - p\left( {y^{i} {|}{\varvec{x}}^{i} , {\mathbf{W}}_{0} ;{\mathbf{W}}_{s1} ,{\varvec{b}}} \right)} \right)$$

where $p\left( {y^{i} {|}{\varvec{x}}^{i} , {\mathbf{W}}_{0} ;{\mathbf{W}}_{s1} ,{\varvec{b}}} \right)$ is the output probability of softmax unit in the 5-layer DNN, given input data ${\varvec{x}}^{i}$ of subject $i$ and initial weights ${\mathbf{W}}_{0}$. ${\varvec{b}}$ represents the bias. A mini-batch stochastic gradient descent algorithm^43^ was used to optimize the objective function. Hyper-parameters were selected based on the successful convergence of the objective function. The learning rate was set as 0.01. We applied an early stop mechanism, which would cease the optimization process if 3 consecutive epochs return the same validation loss errors.

#### Stage 2: unsupervised training in intermediate domain

Given *m* training samples in neonatal dataset from our intermediate cohort, {${\varvec{x}}^{1}$, …, ${\varvec{x}}^{i}$, …,${\varvec{x}}^{m}$}, where ${\varvec{x}}^{i} = \left[ {x_{1}^{i} , \ldots ,x_{n}^{i} } \right]$ is the $n$-dimensional input without label of *i-th* sample. We constructed a 9-layer stacked sparse autoencoder (SSAE) (Fig. 2B), which takes ${\mathbf{W}}_{s1}$ as initial network weights. A rectified linear unit activation function was used in hidden nodes, and a sigmoid unit was chosen in the output layer. We minimized the following cross-entropy objective function to obtain ${\mathbf{W}}_{s2}$,

$$J\left( {{\mathbf{W}}_{s2} ,{\varvec{b}}{|}{\mathbf{W}}_{s1} } \right) = - \frac{1}{mn}\mathop \sum \limits_{i = 1}^{m} \mathop \sum \limits_{j = 1}^{n} {\varvec{x}}_{j}^{i} \log \left( {\hat{\user2{x}}_{j}^{i} \left( {{\mathbf{W}}_{s1} ;{\mathbf{W}}_{s2} ,{\varvec{b}}} \right)} \right) + \left( {1 - {\varvec{x}}_{j}^{i} } \right)\log \left( {1 - \hat{\user2{x}}_{j}^{i} \left( {{\mathbf{W}}_{s1} ;{\mathbf{W}}_{s2} ,{\varvec{b}}} \right)} \right)$$

where $\hat{\user2{x}}_{j}^{i} \left( {{\mathbf{W}}_{s1} ;{\mathbf{W}}_{s2} ,{\varvec{b}}} \right)$ is the reconstructed input ${\varvec{x}}_{j}^{i}$ from *j-th* neuron of the SSAE; ${\varvec{b}}$ represents the bias. A mini-batch stochastic gradient descent algorithm^43^ was used to optimize the objective function and an early stop mechanism was applied. Hyper-parameters were selected based on the successful convergence of objective function. We set a batch size of 16 and a total epoch of 50. The learning rate was set as 0.01.

#### Stage 3: fine-tuning and joint outcome prediction in target domain

Using *m* training sample in preterm dataset from our target cohort, {(${\varvec{x}}^{1} ,\left[ {y_{c}^{1} } \right]_{c = 1}^{C}$), …, (${\varvec{x}}^{i} ,\left[ {y_{c}^{i} } \right]_{c = 1}^{C}$),… ,$({\varvec{x}}^{m} ,[y_{c}^{m} ]_{c = 1}^{C}$)} where ${\varvec{x}}^{i}$ is the *i-th* input of the samples and $y^{i}$ is its corresponding labels of C tasks (i.e., a binary numbers indicating high risk versus low risk of developing neurodevelopmental deficits), we constructed a multi-task DNN (Fig. 2C), which takes ${\mathbf{W}}_{s2}$ as initial network weights and minimized the weighted cross-entropy objective function as follows:

$$J\left( {{\mathbf{W}}_{t} ,{\varvec{b}}{|}{\mathbf{W}}_{s2} } \right) = - \frac{{\alpha^{c} }}{mC}\mathop \sum \limits_{c = 1}^{C} \mathop \sum \limits_{i = 1}^{m} \beta^{i} y_{c}^{i} \log \left( {p\left( { y_{c}^{i} {|}{\varvec{x}}^{i} , {\mathbf{W}}_{s2} ;{\mathbf{W}}_{t} ,{\varvec{b}}} \right)} \right) + \beta^{i} \left( {1 - y_{c}^{i} } \right)\log \left( {1 - p\left( {y_{c}^{i} {|}{\varvec{x}}^{i} , {\mathbf{W}}_{s2} ;{\mathbf{W}}_{t} ,{\varvec{b}}} \right)} \right)$$

where $p\left( { y_{c}^{i} {|}{\varvec{x}}^{i} , {\mathbf{W}}_{s2} ;{\mathbf{W}}_{t} ,{\varvec{b}}} \right)$ is the task c output of the softmax unit of 7-layer DNN, i.e., the probability of sample ${\varvec{x}}^{i}$ being classified as the class $y_{i}^{c}$ label of *c* class. ${\varvec{b}}$ represents the bias. $\alpha^{c}$ is the weight of task c. To mitigate the impact of unbalanced dataset, $\beta^{i}$ is a weight assigned to $i^{th}$ sample, calculated by the ratio of majority and minority class. A mini-batch stochastic gradient descent algorithm^43^ was used to optimize the objective function. Hyper-parameters were selected based on the successful convergence of objective function. The batch size was set as 4 and the number of epochs was set as 10 and an early stop mechanism was applied. The learning rate was set as 0.01.

#### Model validation and assessment

We evaluated the prediction performance of our proposed model through fivefold cross-validation with the metrics of balanced accuracy, sensitivity, specificity, positive likelihood ratio (LR +), false positive rate (FPR), and area under the receiver operating characteristic curve (AUC). We randomly divided the dataset into fivefolds of approximately equal size. We kept the first fold for testing and the model is trained on the remaining fourfolds. The process was repeated 5 times and each time a different fold of the data was used for validation. We computed the average performance across all 5 times. To evaluate performance variability, 50 iterations of fivefold cross-validation were performed. The mean and SD of validation results were calculated over the 50 iterations. The two-sided Student’s t-test was used to assess performance differences between models. A p-value < 0.05 was considered statistically significant. We conducted the validation experiments using Python 3.6, Keras (version: 2.2.4) with Tensorflow (version: 1.10) backend on a computer workstation (256 GB RAM, 2 × Nvidia GTX1080 Ti).

#### Most discriminative functional connections and clinical features

By implementing a feature ranking approach designed for deep learning algorithms, we are able to explore which functional connections the proposed multi-task, multi-stage DTL model learned to be most discriminative of each of the three neurodevelopmental outcomes. More specifically, given *n* ROIs and each model output $$y^{c}$$ of task c, we calculate the partial derivative $$\frac{{\partial y^{c} }}{{\partial a_{i,j} }}$$ , where $$a_{i,j}$$*i* ≠ *j*, $$\in \left[ {1,2, \ldots ,n} \right], j \in \left[ {1,2, \ldots ,n} \right]$$, is connectivity between ROIs *i* and *j*. A higher absolute value of the partial derivative indicates a higher level of the importance for that specific outcome $$y^{c}$$ (i.e., cognitive or language or motor skill) prediction. Similarly, given *m* clinical features, we calculate the partial derivative $$\frac{{\partial y^{c} }}{{\partial e_{i} }}$$ , where $$e_{i}$$$$\in \left[ {1,2, \ldots ,m} \right]$$. We visualized the functional connection by using BrainNet Viewer^44^ and circularGraph^45^.

## Results

### Subjects

In the source cohort, we included 884 (79.5%) of 1,112 subjects from the ABIDE-I database (ages range: 6.5–64 years, median 14.8 years), whose resting-state functional images were processed and functional connectivity maps (e.g., seed-based correlation analyses) were calculated using C-PAC pipeline^36^. After MRI data quality control, excluding the data with largely incomplete brain coverage, high movement peaks, ghosting, and other scanner artifacts, we included 291 (93%) of 313 enrolled neonatal subjects (mean (SD) gestational age at birth 31.2(4.6) weeks; postmenstrual age (PMA) at scan 42.2 (1.3) weeks) in the intermediate cohort; and we included 51 (87.9%) of 58 enrolled very preterm infants in the target cohort. Among these 51 subjects, 33 who have undergone neurodevelopmental assessments at 2 years corrected were included in the target cohort—5 (15%) subjects have high risk of developing severe/moderate cognitive deficits; 7 (21%) subjects have high risk of developing severe/moderate language deficits; and 5 (15%) subjects have high risk of developing severe/moderate motor deficits.

### Multi-stage versus single-stage DTL

In the multi-task joint prediction of cognition, language, and motor deficits, as compared with the optimized single-stage DTL model^29^, our multi-stage DTL model improved balanced accuracy by 7.3% (p < 0.001), 10.9% (p < 0.001), and 7.5% (p < 0.001) and improved the AUC by 0.09 (p < 0.001), 0.03 (p = 0.031) and 0.10 (p < 0.001), respectively, as shown in Table 1. The single-stage models + LRs ranged from 1.4 to 3.5, while the multi-stage models + LRs were higher, ranging from 2.6 to 6.6. The multi-stage model achieved significantly lower FPR than single-stage model on the prediction of abnormal cognitive (p < 0.001) and language (p < 0.001) functions, while a comparable FPR on the prediction of abnormal motor functions (p = 0.394).

**Table 1:** Performance comparison of our proposed multi-task, multi-stage deep transfer learning (DTL) versus multi-task, single-stage DTL models for the joint prediction of abnormal cognitive, language, and motor outcomes at 2 years corrected age in very preterm infants.

Model	Cognition					
	BA (%)	Sen (%)	Spe (%)	LR +	FPR (%)	AUC
Single-stage	74.2 ± 6.1	68.0 ± 6.0	80.3 ± 6.6	3.5 ± 1.6	19.7 ± 6.6	0.77 ± 0.05
Multi-stage	81.5 ± 3.2	74.0 ± 4.9	88.9 ± 3.1	6.6 ± 1.9	11.1 ± 3.1	0.86 ± 0.05

Model	Language					
	BA (%)	Sen (%)	Spe (%)	LR +	FPR (%)	AUC
Single-stage	58.0 ± 4.6	56.0 ± 6.0	60.0 ± 5.6	1.4 ± 0.2	40.0 ± 5.6	0.63 ± 0.05
Multi-stage	68.9 ± 2.3	60.0 ± 4.0	77.8 ± 3.2	2.6 ± 0.3	22.2 ± 3.2	0.66 ± 0.03

Model	Motor					
	BA (%)	Sen (%)	Spe (%)	LR +	FPR (%)	AUC
Single-stage	66.4 ± 4.7	62.0 ± 4.2	70.7 ± 6.6	2.0 ± 0.4	29.3 ± 6.6	0.74 ± 0.04
Multi-stage	73.9 ± 2.4	76.0 ± 4.9	71.7 ± 3.4	2.6 ± 0.2	28.3 ± 3.4	0.84 ± 0.02

BA, balanced accuracy; Sen, sensitivity; Spe, specificity; LR+, likelihood ratio positive; FPR, false positive rate; AUC, area under the receiver operating characteristic curve.

### Multi-task versus single-task prediction

As compared with prediction of each individual outcome separately, our multi-task joint prediction model increased prediction balanced accuracy by 17.3% (p < 0.001), 9.3% (p < 0.001) and 10.2% (p < 0.001) and increased the AUC by 0.11 (p < 0.001), 0.06 (p = 0.002), and 0.12 (p < 0.001) in predicting cognition, language, and motor deficits, respectively (Table 2). The + LRs of single-task models ranged from 1.5 to 2.0, lower than the ones of multi-task models. The multi-task model achieved significantly lower FPR than single-task model in predicting cognitive (p < 0.001) and language (p < 0.001) deficits, while a comparable FPR in predicting motor deficits (p = 0.194).

**Table 2:** Performance comparison of our proposed multi-task, multi-stage deep transfer learning (DTL) versus single-task, multiple-stage DTL models. Multi-task model simultaneously predicts three abnormal neurodevelopmental (cognitive, language and motor) outcomes, while single-task model predicts each individual outcome independently, at 2 years corrected age in very preterm infants. Both models were trained using the proposed multi-stage DTL strategy.

Model	Cognition					
	BA (%)	Sen (%)	Spe (%)	LR +	FPR (%)	AUC
Single-task	64.2 ± 4.3	54.0 ± 6.7	74.4 ± 4.2	2.0 ± 0.3	25.6 ± 4.2	0.75 ± 0.04
Multi-task	81.5 ± 3.2	74.0 ± 4.9	88.9 ± 3.1	6.6 ± 1.9	11.1 ± 3.1	0.86 ± 0.05

Model	Language					
	BA (%)	Sen (%)	Spe (%)	LR +	FPR (%)	AUC
Single-task	59.6 ± 3.5	51.0 ± 8.0	68.2 ± 5.2	1.5 ± 0.2	31.8 ± 5.2	0.60 ± 0.05
Multi-task	68.9 ± 2.3	60.0 ± 4.0	77.8 ± 3.2	2.6 ± 0.3	22.2 ± 3.2	0.66 ± 0.03

Model	Motor					
	BA (%)	Sen (%)	Spe (%)	LR +	FPR (%)	AUC
Single-task	63.7 ± 3.7	54.0 ± 6.3	73.3 ± 4.6	1.9 ± 0.3	26.7 ± 4.6	0.72 ± 0.03
Multi-task	73.9 ± 2.4	76.0 ± 4.9	71.7 ± 3.4	2.6 ± 0.2	28.3 ± 3.4	0.84 ± 0.02

BA, balanced accuracy; Sen, sensitivity; Spe, specificity; LR+, likelihood ratio positive; FPR, false positive rate; AUC, area under the receiver operating characteristic curve.

### Fusion of brain connectome and clinical data versus only clinical data or only brain connectome data

Lastly, we showed in Table 3, that using the fusion of clinical and brain connectome features, our model improved balanced accuracy by 12.4% (p < 0.001), 21.6% (p < 0.001) and 2.2% (p = 0.037), and AUC by 0.07 (p = 0.003), 0.15 (p < 0.001) and 0.12 (p < 0.001) compared with using only clinical features in predicting cognitive, language, and motor deficits, respectively; as well improved the balanced accuracy by 10.6% (P < 0.001), 6.1% (p < 0.001) and 3.8% (p = 0.021) and improved AUC by 0.08 (p = 0.002), 0.06 (p = 0.003) and 0.18 (p < 0.001) compared with using only brain connectome features in predicting cognitive, language, and motor deficits, respectively. On the prediction of motor deficits, no significant FPR differences were observed between the model using combined data and the model using either clinical (p = 0.075) or connectome data (p = 0.056) alone.

**Table 3:** Performance comparison of our proposed multi-task, multi-stage deep transfer learning model using only connectome data versus only clinical data versus combined brain connectome and clinical data, for the joint prediction of abnormal cognitive, language, and motor outcomes at 2 years corrected age in very preterm infants.

Model	Cognition					
	BA (%)	Sen (%)	Spe (%)	LR +	FPR (%)	AUC
Connectome data	70.9 ± 4.5	70.0 ± 6.9	71.7 ± 5.5	2.4 ± 0.4	28.3 ± 5.5	0.78 ± 0.06
Clinical data	69.1 ± 4.3	76.0 ± 4.6	62.1 ± 3.7	2.0 ± 0.2	37.9 ± 3.7	0.79 ± 0.04
Combined data	81.5 ± 3.2	74.0 ± 4.9	88.9 ± 3.1	6.6 ± 1.9	11.1 ± 3.1	0.86 ± 0.05

Model	Language					
	BA (%)	Sen (%)	Spe (%)	LR +	FPR (%)	AUC
Connectome data	62.8 ± 3.7	63.2 ± 6.8	62.4 ± 5.7	1.6 ± 0.2	37.6 ± 5.7	0.60 ± 0.05
Clinical data	47.3 ± 3.2	41.0 ± 3.5	53.6 ± 4.1	0.9 ± 0.1	46.4 ± 4.1	0.51 ± 0.03
Combined data	68.9 ± 2.3	60.0 ± 4.0	77.8 ± 3.2	2.6 ± 0.3	22.2 ± 3.2	0.66 ± 0.03

Model	Motor					
	BA (%)	Sen (%)	Spe (%)	LR +	FPR (%)	AUC
Connectome data	70.1 ± 4.2	72.0 ± 7.3	68.1 ± 5.3	2.2 ± 0.3	31.9 ± 5.3	0.66 ± 0.06
Clinical data	71.7 ± 3.3	74.0 ± 3.6	69.3 ± 3.6	2.4 ± 0.3	30.7 ± 3.6	0.72 ± 0.02
Combined data	73.9 ± 2.4	76.0 ± 4.9	71.7 ± 3.4	2.6 ± 0.2	28.3 ± 3.4	0.84 ± 0.02

BA, balanced accuracy; Sen, sensitivity; Spe, specificity; LR+, likelihood ratio positive; FPR, false positive rate; AUC, area under the receiver operating characteristic curve.

### Most discriminative functional connections and clinical features

**Figure 3:**
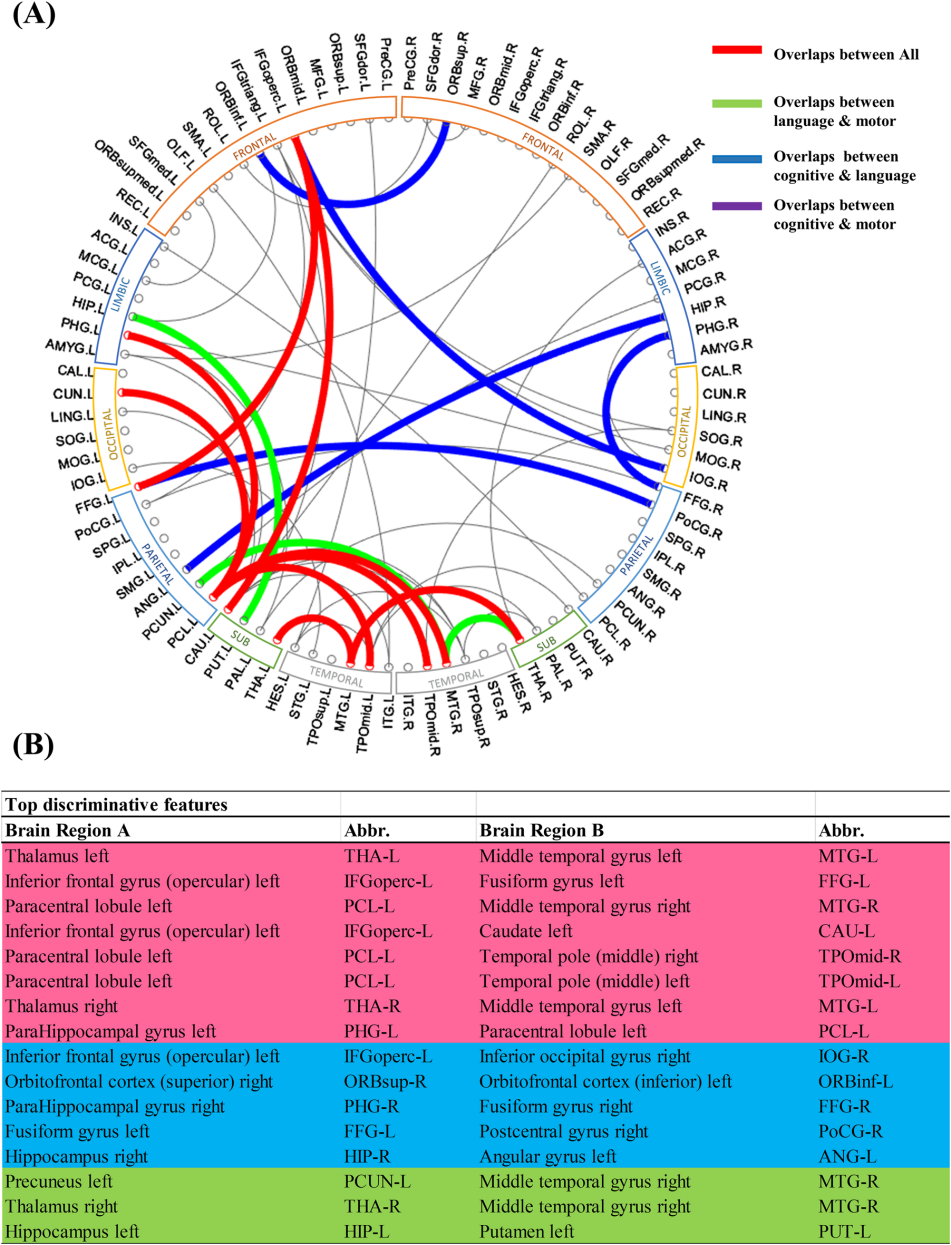
Top discriminative functional connectomes explored by our multi-task, multi-stage deep transfer learning model for each of the three neurodevelopmental outcomes. (**A**) The top 20 functional connections were displayed in the circos plot. (**B**) Overlapped connections were listed. We found 9 common connections predictive to all three outcomes (red), 12 common connections predictive to both language and motor outcomes (green, red); 14 common connections predictive to both cognitive and language outcomes (blue, red); and 9 common connections predictive to both cognitive and motor outcomes (covered by red).

For each prediction task, we identified top 20 most predictive connections. We found 9 common connections predictive to all three neurodevelopmental outcomes (Fig. 3). These regions, including the thalamus, middle temporal gyrus, inferior frontal gyrus, fusiform gyrus, and paracentral gyrus, among others, serve important functions for language, sensory, motor, object vision, and cognitive function. Even a region such as the thalamus, which has been traditionally linked with sensorimotor and language function, was recently identified as important in decision-making and cognitive control^46^. Our findings highlight some of the key functional brain regions that are involved in cognitive, language, and motor development in very preterm infants and further suggests that our proposed model's process of functional connectivity selection for prediction is grounded in well-established brain structure–function relationships. Top discriminative functional connectomes for three neurodevelopmental outcomes are elaborated in Supplemental Table 2.

Our proposed model also selected several known clinical features, including birth weight, gestational age, bronchopulmonary dysplasia, retinopathy of prematurity, maternal socioeconomic status, and maternal magnesium therapy, as important predictors of cognitive, language, and motor deficits at 2 years corrected age. Top discriminative clinical features for three neurodevelopmental outcomes are elaborated in Supplemental Table 3.

## Discussion

In this study, we jointly predicted abnormal neurodevelopmental outcomes at 2 years of age in very preterm infants using neuroimaging and clinical data collected at term-equivalent age. While some studies have shown that neurocognitive development do not stabilize until adolescence, more recent studies have shown that general cognition in extremely preterm infants remains stable from 2 years of age until adolescence^47–49^, and valuable information can still be obtained through standardized cognitive testing at age 24 months corrected age. For example, a cohort study of extremely preterm infants showed that standardized cognitive scores at 24 months corrected age explained 38% of the variance in IQ scores at age 8–9 in extremely preterm infants^50^. This was higher than the variance explained by scores at 18 months corrected age.

Current tools to predict neurodevelopmental outcomes of very preterm infants are limited^51–53^. Studies from the National Institute of Child Health and Human Development (NICHD) Neonatal Research Network have attempted to develop models based on five risk factors including gestational age, sex, birth weight, antenatal steroids, and plurality^54–56^. However, clinical data to predict neurodevelopmental outcomes have only achieved modest prediction accuracy with an area under the curve (AUC) of 0.68^56^. Therefore, there is a need to examine the incremental value of brain MRI biomarkers over clinical factors^57–61^.

Some recent studies have investigated the prediction power of connectome features derived from diffusion tensor images (DTI). For example, Kawahara et al.^62^, developed a convolutional neural network for brain network (BrainNetCNN) using DTI-derived structural brain connectome of preterm infants to predict Bayley-III cognitive and motor scores, assessed at 18 months of age. The Pearson’s correlation coefficients between the actual cognitive and motor scores, and the ones predicted by BrainNetCNN are 0.188 and 0.310, respectively. Additionally, Girault et al.^63^, proposed a DNN model using structural brain connectome of neonates at term-equivalent age to predict Early Learning Composite (ELC) standardized score at 2 years of age. Predicted ELC scores by the DNN model were significantly correlated with actual ELC scores with a Pearson’s correlation coefficient of 0.98 for full-term infants, and 0.96 for preterm infants. Other studies have found that DTI-based features, such as fractional anisotropy (FA) are correlated with neurodevelopmental outcomes of preterm infants^64,65^. Most recently, Saha et al.^66^, proposed a deep CNN model using FA data to identify infants with abnormal motor outcome (measured by Neuro-Sensory Motor Developmental Assessment) at 2 years and achieved an AUC of 0.72 (SD 0.14) and an accuracy of 73% (SD 19%).

In addition, brain functional connectome studies in adults and older children have shown that abnormal network properties may be useful as discriminative features for early diagnosis in a variety of neurological conditions, such as attention deficit hyperactivity disorder^67^, and autism spectrum disorder^28^. We previously demonstrated that an artificial neural network model applied to functional connectome data can identify very preterm infants at high-risk for cognitive deficits at 2 years of corrected age with an accuracy of 70.6% (SD 4.9%) and an AUC of 0.76 (SD 0.03)^29^, which is significantly lower than the performance of our current presented deep transfer learning model with an accuracy of 81.5% (SD 3.2%) and an AUC of 0.86 (SD 0.05). Although the final + LRs achieved for the language and motor models are not of clinical use, inspiringly, for prediction of cognitive deficits, our multi-task, multi-stage model did show a promising LR + of 6.7. Nevertheless, a larger study is important to validate our approach to further assess its clinical utility.

To mitigate concerns regarding insufficient data for training a deep learning model, we employed transfer learning technique. Unlike previous work, instead of directly transferring the knowledge of brain connectome patterns learned from ABIDE-I, we here introduced an intermediate domain (i.e., neonatal brain connectome) to bridge the distribution gap between brain connectome of older children and adults and very preterm infants. We proposed to learn brain connectome patterns from very preterm infants with the aid of first learning from older children and adults and then customizing this prior-knowledge via learning from neonatal infants. We also improved on our previous work by incorporating clinical data and by simultaneously predicting multiple relevant neurodevelopmental (cognitive, language and motor) outcomes rather than only one.

Our study has limitations. First, our current brain functional connectome analysis was based on an anatomical/structural atlas rather than a functional brain parcellated atlas^68^, therefore our functional connectivity estimation could be affected by within-ROI signal heterogeneity. Second, neurodevelopmental outcomes for those 291 neonatal subjects are not currently available. We expect better performance if we could include annotated neonatal data for supervised training. Third, although 884 subjects and 291 neonatal subjects that we used for pre-training are considered large numbers in this field of clinical research, larger datasets are preferred to validate the concept of multi-stage DTL. Finally, besides the brain functional connectome we used, neurodevelopmental outcome prediction may be further improved by also incorporating the brain structural connectome, derived from diffusion MRI, as we are currently undertaking.

In summary, we presented a multi-task, multi-stage DTL using the fusion of brain connectome and clinical data for early joint prediction of abnormal neurodevelopmental outcomes at 2 years of age in very preterm infants. We first supervisedly pre-trained a DNN prototype using 884 ASD patients and control subjects (with annotated labels), and then re-trained this DNN prototype using 291 neonatal subjects (without neurodevelopmental assessments; i.e. without annotated labels). Finally, we fine-tuned and validated the DNN model using 33 very preterm infants (with neurodevelopmental assessments). We demonstrated that the knowledge gained from classifying ASD patients versus typically developing controls in children and adults can be transferred to predict neurodevelopmental deficits in infants. The model performance was evaluated by comparing with (1) single-stage DTL model; (2) signal-task model; and (3) using clinical or brain connectome data only. The key findings of this study can be summarized as: (1) multi-stage DTL strategy maximizes the value of both annotated and non-annotated data in model training by performing both supervised and unsupervised learning. It outperforms single-stage DTL model; (2) simultaneous prediction of multiple outcomes (i.e., learning related tasks jointly) improves performance as compared with prediction of single outcome independently (i.e., learning each task individually); and (3) using the fusion of brain connectome and clinical data markedly improves prediction performance over using either clinical data only or brain connectome data only. Employing such a deep learning model, once externally validated, may facilitate risk stratification at term-equivalent age for early identification of long-term neurodevelopmental deficits and facilitate targeted early interventions to improve clinical outcomes in very preterm infants.

## Supplementary information

Supplementary information.
