# Supplementary material for: A multi-task, multi-stage deep transfer learning model for early prediction of neurodevelopment in very preterm infants
Source: Sci Rep. 2020 Sep 15;10:15072. doi: 10.1038/s41598-020-71914-x (PMC7492237; doi:10.1038/s41598-020-71914-x)
Supplement: Supplementary file 1 — Supplementary information. [file 41598_2020_71914_MOESM1_ESM.docx]

**Supplemental Table 1:** Full list of 84 clinical features for very preterm infants in the target cohort.

| **Category** | **Index** | **Variable name** | **Range/Proportion** | **Note** |
| --- | --- | --- | --- | --- |
| **Material information** | 1 | Mothers age (years) | Range:[17,48], Median: 32 |  |
|  | 2 | Gravida | Range:[1,8], Median: 2 |  |
|  | 3 | Parity | Range:[1,5], Median: 2 |  |
|  | 4 | Marital status | 24 (72.7%) | Married |
|  | 5 | Highest education level | Range:[2,7], Median: 5 | 1. <7th grade,2. 7th to 9th grade,3. 10th to 12th grade,4. High School degree,5. Partial college,6. College degree,7. Graduate degree,8. Unknown |
|  | 6 | Maternal annual income | Range:[1,9], Median: 6 | 1. less than $10k, 2. $10k- $19k, 3. $20k - $29k, 4. $30k - $39k, 5. $40k - $59k, 6. $60k - $79k, 7. $80k - $99k, 8. More than 100k. 9. Prefer not to answer |
|  | 7 | Maternal smoking status | 12 (36.4%) | Yes |
|  | 8 | Street drugs during pregnancy | 1 (3%) | Yes |
|  | 9 | Narcotics during pregnancy | 29 (87.9%) | No |
| **Pregnancy complications** | 10 | Multiple birth | 10 (30.3%) | Yes |
|  | 11 | Diabetes insulin | 1 (3%) | Yes |
|  | 12 | Hypertension | 11 (33.3%) | Yes |
|  | 13 | Hyperthyroidism | 3 (9.1%) | Yes |
|  | 14 | Antepartum hemorrhage | 5 (15.2%) | Yes |
|  | 15 | Chorioamnionitis | 6 (18.2%) | Yes |
|  | 16 | Placental pathology | 9 (27.3%) | Yes |
|  | 17 | Fertility treatment | 10 (30.3%) | Yes |
| **Labor and delivery** | 18 | Rupture of membrane | 12 (36.4%) | Yes |
|  | 19 | Steroids | 32 (97%) | Yes |
|  | 20 | Magnesium | 21 (63.6%) | Yes |
|  | 21 | Antibiotics | 26 (78.8%) | Yes |
|  | 22 | Delivery mode | 8 (24.2%) | Yes |
|  | 23 | Contractions | 16 (48.5%) | Yes |
|  | 24 | Infections history | 8 (24.2%) | Yes |
|  | 25 | Fever during labor | 2 (6.1%) | Yes |
| **Neonatal information at birth** | 26 | Birth hospital | Range:[1,4], Median: 1 | 1. OSU,2. RMH,3. SA, 4. Other |
|  | 27 | Clamping delay >30 sec | 7 (21.2%) |  |
|  | 28 | Umbilical cord milked prior to clamping | 30 (90.9%) |  |
|  | 29 | Infant sex | 17 (51.5%) | Male |
|  | 30 | Ethnic categories | 33 (100%) | Not Hispanic or Latino |
|  | 31 | Racial categories | Range:[1,6], Median: 2 | 1. Black,2. White,3. American Indian or Alaskan Native,4. Asian,5. Native Hawaiian or other Pacific Islander,6. More Than One Race,7. Unknown or Not Reported |
|  | 32 | Gestational age at birth (total weeks) | Range:[23.7,31.9], Median: 28.6 |  |
|  | 33 | Gestational age at birth (total days) | Range:[168,229], Median: 204 |  |
|  | 34 | Oxygen support at birth | 30 (90.9%) | Yes |
|  | 35 | Positive pressure ventilation at birth | 21 (63.6%) | Yes |
|  | 36 | CPAP at birth | 24 (72.7%) | Yes |
|  | 37 | Intubation at birth | 8 (24.2%) | Yes |
|  | 38 | Chest compression at birth | 1 (3%) | Yes |
|  | 39 | Birth weight (grams) | Range:[510,1900], Median: 1135 |  |
|  | 40 | Birth length (cm) | Range:[28,43.5], Median: 38.5 |  |
|  | 41 | Head circumference at birth (cm) | Range:[19.5,30], Median: 26 |  |
|  | 42 | Cord blood gas at birth | 3 (9.1%) | Yes |
| **Neonatal information at term-equivalent age** | 43 | Status of infant at follow-up | Range:[1,2], Median: 1 | 1 = Discharged to home , 2 = Still in hospital at 40 weeks PMA, 3 = Transferred to another hospital, 4 = Transferred to chronic care facility, 5 = Death |
|  | 44 | Weight at follow-up (grams) | Range:[2055,3925], Median: 2970 |  |
|  | 45 | Length at follow-up (cm) | Range:[42.5,51.5], Median: 46.5 |  |
|  | 46 | Head circumference at follow-up (cm) | Range:[30,36], Median: 33.2 |  |
|  | 47 | Gestational age at follow-up (total weeks) | Range:[34.1,40], Median: 39.6 |  |
|  | 48 | Gestational age at follow-up (total days) | Range:[240,285], Median: 280 |  |
|  | 49 | Referral to child protective services | 31 (93.9%) | No |
| **Medical history** | 50 | Oxygen or positive pressure support | 2 (6.1%) | No |
|  | 51 | Surfactant | 19 (57.6%) | No |
|  | 52 | Pneumothorax | 32 (97%) | No |
|  | 53 | Pulmonary hemorrhage | 33 (100%) | No |
|  | 54 | Steroids for BPD/CLD | 32 (97%) | No |
|  | 55 | Total number of days on oxygen therapy | Range:[0,114], Median: 41 |  |
|  | 56 | Total number of days on CPAP | Range:[0,68], Median: 31 |  |
|  | 57 | Total number of days on ventilation therapy | Range:[0,93], Median: 0 |  |
|  | 58 | Total number of days on HFOV | Range:[0,17], Median: 0 |  |
|  | 59 | Respiratory support type at 36-week | 15 (45.5%) | No |
|  | 60 | Pulmonary hypertension history | 31 (93.9%) | No |
|  | 61 | Patent ductus arteriosus history | 28 (84.8%) | No |
|  | 62 | Chest compressions history | 31 (93.9%) | No |
|  | 63 | Indomethacin history for prophylaxis | 29 (87.9%) | No |
|  | 64 | Seizure history | 33 (100%) | No |
|  | 65 | Cranial sonograms history before 35 weeks PMA | 4 (12.1%) | No |
|  | 66 | Cranial sonograms history at or after 35 weeks PMA | 9 (27.3%) | No |
|  | 67 | PMA at scan (total weeks) | Range:[39.3,41.4], Median: 40.3 |  |
|  | 68 | PMA at scan (total days) | Range:[277,293], Median: 284 |  |
|  | 69 | Early onset septicemia/bacteremia | 32 (97%) | No |
|  | 70 | Late onset culture positive septicemia/bacteremia | 30 (90.9%) | No |
|  | 71 | Meningitis | 32 (97%) | No |
|  | 72 | Parenteral alimentation (total days) | Range:[5,80], Median: 14 |  |
|  | 73 | Breast milk in the first 28 days | 31 (93.9%) | No |
|  | 74 | Necrotizing enterocolitis | 31 (93.9%) | No |
|  | 75 | GI surgery that resulted in short gut | 31 (93.9%) | No |
|  | 76 | PRBC transfusions | 17 (51.5%) | No |
|  | 77 | Caffeine for apnea/neuroprotection | 4 (12.1%) | No |
|  | 78 | Iron supplementation | 2 (6.1%) | No |
|  | 79 | Hearing screen | 4 (12.1%) | No |
|  | 80 | Retinopathy of prematurity exam | 3 (9.1%) | No |
|  | 81 | Retinopathy of prematurity status | Range:[1,3], Median: 3 | 1 = Determined, favorable in both eyes, 2 = Determined, severe ROP in either eye, 3 = Undetermined ROP status in either eye (and neither had “severe ROP”) |
|  | 82 | Major surgery | 29 (87.9%) | No |
|  | 83 | Syndromes and/or major malformations | 33 (100%) | No |
|  | 84 | Status of infant at 36-week | Range:[1,2], Median: 2 | 1 = Discharged to home , 2 = Still in hospital at 40 weeks PMA, 3 = Transferred to another hospital, 4 = Transferred to chronic care facility, 5 = Death |

**Supplemental Table 2:** Top discriminative functional connectomes explored by our multi-task, multi-stage deep transfer learning model for three neurodevelopmental outcomes, respectively.

| **Cognition** | | | |
| --- | --- | --- | --- |
| **Node A** | | **Node B** | |
| Thalamus left | THA-L | Middle temporal gyrus left | MTG-L |
| Inferior frontal gyrus (opercular) left | IFGoperc-L | Fusiform gyrus left | FFG-L |
| Paracentral lobule left | PCL-L | Middle temporal gyrus right | MTG-R |
| Inferior frontal gyrus (opercular) left | IFGoperc-L | Inferior occipital gyrus right | IOG-R |
| Inferior frontal gyrus (opercular) left | IFGoperc-L | Caudate left | CAU-L |
| Paracentral lobule left | PCL-L | Temporal pole (middle) right | TPOmid-R |
| Paracentral lobule left | PCL-L | Temporal pole (middle) left | TPOmid-L |
| Thalamus right | THA-R | Middle temporal gyrus left | MTG-L |
| ParaHippocampal gyrus left | PHG-L | Paracentral lobule left | PCL-L |
| Orbitofrontal cortex (superior) right | ORBsup-R | Orbitofrontal cortex (inferior) left | ORBinf-L |
| ParaHippocampal gyrus right | PHG-R | Fusiform gyrus right | FFG-R |
| Fusiform gyrus left | FFG-L | Postcentral gyrus right | PoCG-R |
| Olfactory left | OLF-L | Anterior cingulate gyrus left | ACG-L |
| Hippocampus right | HIP-R | Angular gyrus left | ANG-L |
| Inferior frontal gyrus (opercular) left | IFGoperc-L | Middle occipital gyrus right | MOG-R |
| Supplementary motor area left | SMA-L | Temporal pole (middle) left | TPOmid-L |
| Cuneus left | CUN-L | Paracentral lobule left | PCL-L |
| Precentral gyrus left | PreCG-L | Thalamus left | THA-L |
| Orbitofrontal cortex (superior) right | ORBsup-R | Orbitofrontal cortex (middle) right | ORBmid-R |
| Middle occipital gyrus left | MOG-L | Heschl gyrus left | HES-L |
|  |  |  |  |
| **Language** | | | |
| **Node A** | | **Node B** | |
| Inferior frontal gyrus (opercular) left | IFGoperc-L | Fusiform gyrus left | FFG-L |
| Thalamus left | THA-L | Middle temporal gyrus left | MTG-L |
| Paracentral lobule left | PCL-L | Middle temporal gyrus right | MTG-R |
| Inferior frontal gyrus (opercular) left | IFGoperc-L | Caudate left | CAU-L |
| Paracentral lobule left | PCL-L | Temporal pole (middle) right | TPOmid-R |
| Thalamus right | THA-R | Middle temporal gyrus left | MTG-L |
| Paracentral lobule left | PCL-L | Temporal pole (middle) left | TPOmid-L |
| Inferior frontal gyrus (opercular) left | IFGoperc-L | Inferior occipital gyrus right | IOG-R |
| ParaHippocampal gyrus left | PHG-L | Paracentral lobule left | PCL-L |
| Hippocampus right | HIP-R | Angular gyrus left | ANG-L |
| Paracentral lobule right | PCL-R | Putamen left | PUT-L |
| ParaHippocampal gyrus right | PHG-R | Fusiform gyrus right | FFG-R |
| Cuneus left | CUN-L | Paracentral lobule left | PCL-L |
| ParaHippocampal gyrus right | PHG-R | Supramarginal gyrus left | SMG-L |
| Precuneus left | PCUN-L | Middle temporal gyrus right | MTG-R |
| Middle occipital gyrus right | MOG-R | Inferior temporal gyrus right | ITG-R |
| Fusiform gyrus left | FFG-L | Postcentral gyrus right | PoCG-R |
| Orbitofrontal cortex (superior) right | ORBsup-R | Orbitofrontal cortex (inferior) left | ORBinf-L |
| Thalamus right | THA-R | Middle temporal gyrus right | MTG-R |
| Hippocampus left | HIP-L | Putamen left | PUT-L |
|  |  |  |  |
| **Motor** | | | |
| **Node A** | | **Node B** | |
| Inferior frontal gyrus (opercular) left | IFGoperc-L | Fusiform gyrus left | FFG-L |
| Thalamus left | THA-L | Middle temporal gyrus left | MTG-L |
| Paracentral lobule left | PCL-L | Middle temporal gyrus right | MTG-R |
| Paracentral lobule left | PCL-L | Temporal pole (middle) right | TPOmid-R |
| Paracentral lobule left | PCL-L | Temporal pole (middle) left | TPOmid-L |
| Inferior frontal gyrus (opercular) left | IFGoperc-L | Caudate left | CAU-L |
| Thalamus left | THA-L | Middle temporal gyrus right | MTG-R |
| Thalamus right | THA-R | Middle temporal gyrus right | MTG-R |
| Thalamus right | THA-R | Middle temporal gyrus left | MTG-L |
| Hippocampus left | HIP-L | Fusiform gyrus right | FFG-R |
| Cuneus left | CUN-L | Paracentral lobule left | PCL-L |
| Olfactory right | OLF-R | ParaHippocampal gyrus left | PHG-L |
| Supplementary motor area right | SMA-R | Paracentral lobule left | PCL-L |
| Putamen right | PUT-R | Middle temporal gyrus left | MTG-L |
| Rectus gyrus left | REC-L | Caudate right | CAU-R |
| Precuneus left | PCUN-L | Middle temporal gyrus right | MTG-R |
| ParaHippocampal gyrus left | PHG-L | Paracentral lobule left | PCL-L |
| Inferior frontal gyrus (triangular) left | IFGtriang-L | Posterior cingulate gyrus left | PCG-L |
| Hippocampus left | HIP-L | Putamen left | PUT-L |
| Middle cingulate gyrus right | MCG-R | Heschl gyrus right | HES-R |

**Supplemental Table 3:** Top discriminative clinical features ranked by our multi-task, multi-stage deep transfer learning model for three neurodevelopmental outcomes, respectively.

| **Cognition** |  | **Language** |  | **Motor** |
| --- | --- | --- | --- | --- |
| **Features** |  | **Features** |  | **Features** |
| Birth weight (grams) |  | Surfactant history |  | Birth weight (grams) |
| Surfactant history |  | Birth weight (grams) |  | Surfactant history |
| Antibiotics |  | Antibiotics |  | Antibiotics |
| Gestational age at birth (total days) |  | Gestational age at birth (total days) |  | Hearing screen history |
| Antepartum hemorrhage |  | Hearing screen history |  | Gestational age at birth (total days) |
| Hearing screen history |  | Antepartum hemorrhage |  | Antepartum hemorrhage |
| Magnesium |  | Maternal smoking status |  | Magnesium |
| Maternal smoking status |  | Retinopathy of prematurity status |  | Retinopathy of prematurity status |
| Hypertension |  | Hypertension |  | Maternal smoking status |
| Retinopathy of prematurity status |  | Magnesium |  | Hypertension |
| Steroids for BPD/CLD |  | Cord blood gas at birth |  | Chest compressions history |
| Status of infant at follow-up |  | Rupture of membrane |  | Steroids for BPD/CLD |
| Rupture of membrane |  | Maternal annual income |  | Caffeine for apnea/neuroprotection |
| Positive pressure ventilation at birth |  | Length at follow-up (cm) |  | Birth length (cm) |
| Respiratory support type at 36-week |  | Status of infant at follow-up |  | Status of infant at follow-up |
| Chest compressions history |  | Steroids for BPD/CLD |  | Positive pressure ventilation at birth |
| Maternal annual income |  | Respiratory support type at 36-week |  | Respiratory support type at 36-week |
| Cord blood gas at birth |  | Pulmonary hypertension history |  | Rupture of membrane |
| Birth length (cm) |  | Positive pressure ventilation at birth |  | Maternal annual income |
| Necrotizing enterocolitis |  | Pulmonary hemorrhage |  | Necrotizing enterocolitis |
